# Unexpected decline in tuberculosis cases coincident with economic recession -- United States, 2009

**DOI:** 10.1186/1471-2458-11-846

**Published:** 2011-11-07

**Authors:** Carla A Winston, Thomas R Navin, Jose E Becerra, Michael P Chen, Lori R Armstrong, Carla Jeffries, Rachel S Yelk Woodruff, Jessie Wing, Angela M Starks, Craig M Hales, J Steve Kammerer, William R Mac Kenzie, Kiren Mitruka, Mark C Miner, Sandy Price, Joseph Scavotto, Ann M Cronin, Phillip Griffin, Philip A LoBue, Kenneth G Castro

**Affiliations:** 1 Division of Tuberculosis Elimination, National Center for HIV/AIDS, Viral Hepatitis, STD, and TB Prevention, Centers for Disease Control and Prevention, 1600 Clifton Road NE, Atlanta, GA, 30333 USA; 2 Northrop Grumman Information Systems, 3375 Northeast Expressway NE, Atlanta, GA, 30333, USA; 3 Public Health Surveillance Program Office, Division of Health Information, Office of Surveillance Epidemiology and Laboratory Services, Centers for Disease Control and Prevention, 1600 Clifton Road NE, Atlanta, GA, 30333, USA; 4 Tuberculosis Control Program, Kansas Department of Health and Environment, 1000 SW Jackson Street, Topeka, KS, 66612, USA, and Immediate Past President, National Tuberculosis Controllers Association, 2452 Spring Rd SE, Smyrna, GA, 30080, USA

## Abstract

**Background:**

Since 1953, through the cooperation of state and local health departments, the U.S. Centers for Disease Control and Prevention (CDC) has collected information on incident cases of tuberculosis (TB) disease in the United States. In 2009, TB case rates declined -11.4%, compared to an average annual -3.8% decline since 2000. The unexpectedly large decline raised concerns that TB cases may have gone unreported. To address the unexpected decline, we examined trends from multiple sources on TB treatment initiation, medication sales, and laboratory and genotyping data on culture-positive TB.

**Methods:**

We analyzed 142,174 incident TB cases reported to the U. S. National Tuberculosis Surveillance System (NTSS) during January 1, 2000-December 31, 2009; TB control program data from 59 public health reporting areas; self-reported data from 50 CDC-funded public health laboratories; monthly electronic prescription claims for new TB therapy prescriptions; and complete genotyping results available for NTSS cases. Accounting for prior trends using regression and time-series analyses, we calculated the deviation between observed and expected TB cases in 2009 according to patient and clinical characteristics, and assessed at what point in time the deviation occurred.

**Results:**

The overall deviation in TB cases in 2009 was -7.9%, with -994 fewer cases reported than expected (*P *< .001). We ruled out evidence of surveillance underreporting since declines were seen in states that used new software for case reporting in 2009 as well as states that did not, and we found no cases unreported to CDC in our examination of over 5400 individual line-listed reports in 11 areas. TB cases decreased substantially among both foreign-born and U.S.-born persons. The unexpected decline began in late 2008 or early 2009, and may have begun to reverse in late 2009. The decline was greater in terms of case counts among foreign-born than U.S.-born persons; among the foreign-born, the declines were greatest in terms of percentage deviation from expected among persons who had been in the United States less than 2 years. Among U.S.-born persons, the declines in percentage deviation from expected were greatest among homeless persons and substance users. Independent information systems (NTSS, TB prescription claims, and public health laboratories) reported similar patterns of declines. Genotyping data did not suggest sudden decreases in recent transmission.

**Conclusions:**

Our assessments show that the decline in reported TB was not an artifact of changes in surveillance methods; rather, similar declines were found through multiple data sources. While the steady decline of TB cases before 2009 suggests ongoing improvement in TB control, we were not able to identify any substantial change in TB control activities or TB transmission that would account for the abrupt decline in 2009. It is possible that other multiple causes coincident with economic recession in the United States, including decreased immigration and delayed access to medical care, could be related to TB declines. Our findings underscore important needs in addressing health disparities as we move towards TB elimination in the United States.

## Background

Based on National Tuberculosis Surveillance System (NTSS) provisional data in March 2010, the Centers for Disease Control and Prevention (CDC) observed that the decline in reported TB case count in the United States in 2009 was considerably steeper than in recent years [1]. TB case rates declined -11.4% in 2009 compared to an average annual -3.8% decline since 2000. Working with internal and external partners, CDC conducted epidemiologic assessments to explore the substantial and unexpected TB decline. We sought to determine if the decline in TB was real, or whether the decline reflected changes in surveillance reporting or diagnosis. We examined evidence that the decline might be due to population changes, improved TB control, or less transmission of TB.

We investigated the extent to which the decline in reported TB might be the result of recent revisions to the NTSS. Health departments in the 50 states and Washington, D.C. electronically report verified cases of TB to the NTSS according to a standard case definition. In 2009, the national TB case definition [2] was modified. Compared with the 1996 version [3], the updated case definition incorporates newer diagnostic tests. Updates to the case definition were coordinated with revisions in the Report of Verified Case of Tuberculosis, the standardized national data collection instrument used to report data to CDC [4]. Major changes to software systems used to report cases electronically to CDC occurred in 2009; specifically, reporting changed from use of a single CDC software, the Tuberculosis Information Management System, to a choice of any software application (including CDC-developed, vendor-developed, and state-developed systems) that is compliant with U.S. National Electronic Disease Surveillance System standards. An additional concern was whether the United States recession from December 2007 through June 2009 [5] might have led to underreporting of TB to CDC, for instance, if routine surveillance could not be conducted due to health department staff reductions. Declines might reflect fewer patients seeking medical care, or physicians considering the diagnosis of TB less often. We also considered the effect of demographic changes in immigration, and of revised overseas TB screening requirements for U.S. immigration applicants that began to be implemented globally in 2007 and may have increased TB diagnoses and treatment prior to immigration, leading to fewer TB diagnoses among immigrants after U.S. entry [6]. Finally, we considered whether the decline might reflect a decrease in TB transmission by examining TB genotype clustering, because localized clustering is assumed to represent recent transmission [7,8].

## Methods

### Study population

TB cases were analyzed for the time period January 1, 2000, through December 31, 2009, as reported by the 50 states and Washington, D.C., to the NTSS, with data updated through August 27, 2010. NTSS data consist of annual incident cases of TB disease verified according to laboratory or clinical criteria [2,3], and include the month of TB diagnosis verification by public health departments and of TB treatment initiation, patient demographics (age, self-reported race, ethnicity [Hispanic or non-Hispanic], country of origin, social risk factors for TB), and clinical information [9]. Date of symptom onset is not available in the NTSS. Patient insurance status and socioeconomic status are also not available. We analyzed data for foreign-born persons by length of time in the United States and by country of birth. U.S.- or foreign-born designation followed U.S. Census definitions [10].

We collaborated with U.S. TB control officials to validate all individual-level TB case reports for 2008 and 2009 at the county, state, and national level for 10 counties and one large city that together account for one-quarter of all TB cases in the United States (23% in 2008). These areas also provided sentinel information by reporting culture-positive cases of *Mycobacterium tuberculosis *in the first quarter of 2010 for comparison with 2008 and 2009. We assessed the findings from a survey in March 2010 sponsored by the National Tuberculosis Controllers Association, which solicited potential evidence of changes in surveillance or clinical practice from TB jurisdictions, and we analyzed self-reported data as of August 20, 2010 from 50 CDC-funded public health laboratories (45 states, 3 large cities, Washington D.C., and Puerto Rico) to determine the number of suspected TB patients from whom a specimen was submitted for testing and the percentage that were culture-positive for *M. tuberculosis *complex in 2008 and 2009. To assess trends in TB therapy initiation, we analyzed monthly claims for new combined prescriptions of isoniazid, rifamycins, and pyrazinamide, from January 2004 through March 2010 from CDC BioSense [11], which receives RelayHealth™ electronic prescription claims for 20,000-30,000 outpatient pharmacies in 50 states and Washington, D.C.. For clustering, we studied all cases reported to the NTSS with complete genotyping results by spoligotyping and 12-locus mycobacterial interspersed repetitive units analysis [12] with TB treatment start dates January 2007 through December 2009. Cases were considered clustered if their *M. tuberculosis *genotype exactly matched another case in the same county in the same year. We calculated clustering percentages by dividing the number of clustered cases by the total cases with genotyping results.

### Analyses

Using NTSS data, we tabulated TB observed case counts for 2009 according to patient characteristics. We log-transformed counts for years 2000 through 2008, and performed linear regression to calculate expected cases and 95% prediction intervals (PI) for 2009. We calculated the difference between expected and observed 2009 cases, and described the percentage deviation as the difference divided by expected cases, multiplied by 100. We used a Taylor-series approximation to calculate variance for determining *P *values, which were evaluated for hypothesis generation in conjunction with the magnitude of the deviation and prediction interval bounds. We obtained population denominators from the U.S. Census Bureau Current Population Survey annual March supplement [10] and calculated expected TB case rates and 95% PI using Poisson regression.

**Figure 1:**
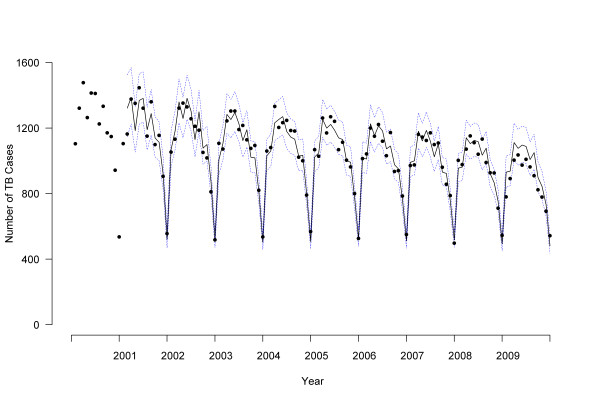
**Observed tuberculosis cases compared to forecast by treatment start date, 2000-2009, United States**. Dots indicate observed monthly tuberculosis case counts. Solid line indicates autoregressive integrated moving average (ARIMA) model for 2000-2007 with forecasting for 2008-2009. Dotted line indicates 95% confidence interval.

To investigate detailed changes over time, monthly NTSS data for TB treatment start date were used. Because of the seasonal pattern of the data, we constructed autoregressive integrated moving average (ARIMA) models for time series. We used 2000-2007 data to estimate ARIMA parameters and forecast expected monthly counts for 2008 and 2009. ARIMA models were constructed (p,d,q), (p,d,q) _12 _to account for first-order (monthly) and second-order (yearly) components for auto-regressive (p), integrated (d), and moving average (q) parameters respectively; the observed data and best-fitting model specified as (0,1,1)(0,1,1)_12 _are shown in Figure 1. We examined differences between observed and expected cases by *P *values, where *P *< .05 was interpreted as a significant difference. Similarly, we analyzed monthly BioSense TB medication pharmacy claims using ARIMA models. Since the ARIMA model forecasted a moving average, the first data point reflected the second year of available data for all analyses (2001 for NTSS, 2005 for BioSense). We used the Akaike information criterion and Schwarz criterion as well as examination of autocorrelation and residual plots to evaluate goodness of fit and select best models. Final model fit was assessed using *R*^2 ^as the coefficient of determination.

We used chi-square statistics for categorical comparisons such as the percentage of patients reported positive for *M. tuberculosis *complex by public health laboratories in 2008 versus 2009 and the percentages of genotype clustering by year. Categorical comparisons, regression models, and ARIMA analyses were conducted in SAS (version 9.2; SAS Institute, Cary, NC). We used monthly ARIMA values to fit Joinpoint linear regression models, from which we identified changes in time trends that were significant at *P *< .05. Joinpoint is a statistical software for analysis of trends that starts by assuming no changes in trend, and uses a Monte Carlo permutation method to determine the number of significant flexion points, or changes in trend (Joinpoint version 3.3; Joinpoint, Bethesda, MD) [13].

## Results

### Decline in TB cases in 2009

In 2009, a total of 11,545 TB cases were reported in the United States. The percentage difference between observed and expected 2009 cases was -7.9%, with -994 fewer cases reported in 2009 than expected (Table 1). The deviation from expected case counts was significant among both U.S.-born (-7.1%) and foreign-born (-11.1%) individuals, and was greatest among foreign-born persons who had been in the United States < 2 years before TB diagnosis (-23.5%). Case counts declined -11.6% among foreign-born persons from countries that had updated TB screening procedures for immigration by adding sputum culture for high-risk persons and tuberculin skin testing for children [6], compared with -10.7% among persons from countries not yet implementing the changes. Of the top 10 countries of origin of foreign-born TB patients, two countries had significant declines: Mexico (-14.8%), which updated TB screening procedures for immigration in 2007, and Guatemala (-27.0%), which has not yet begun to implement updated procedures. Demographic strata with significant declines included Hispanics, non-Hispanic blacks, and adults under age 65 (Table 1). TB declines among homeless persons and injecting drug users were -17.8% and -29.6% greater than expected; these declines were predominantly among U.S.-born TB patients.

**Table 1:** Observed and expected tuberculosis case counts by select demographic and risk factor characteristics for January-December 2009, United States

	Observed Case Count	Expected Case Count (95% Prediction Interval)	Case Count Difference*	Percentage of Expected	*P *value
**Total**	11545	12539 (12233 - 12853)	-994	-7.9	<.001

**Country of origin**					

Foreign-born	6854	7709 (7318 - 8120)	-855	-11.1	<.001

U.S.-born	4571	4918 (4661 - 5190)	-347	-7.1	.005

Unknown	120	21 (10 - 48)	99	471.4	.020

**For foreign-born, length of stay in United States**					

< 2 years	1432	1872 (1590 - 2204)	-440	-23.5	<.001

> = 2 years	4712	5197 (4865 - 5551)	-485	-9.3	.001

Unknown	710	684 (599 - 781)	26	3.8	.587

**For foreign-born, country of origin** **according to implementation of 2007** **immigration technical instructions (TI)**					

TI countries	2993	3384 (3095 - 3699)	-391	-11.6	.002

Non-TI countries	3861	4325 (4044 - 4625)	-464	-10.7	<.001

**Race/ethnicity**					

Hispanic	3380	3953 (3563 - 4385)	-573	-14.5	<.001

Non-Hispanic American Indian	102	121 (100 - 148)	-19	-15.7	.151

Non-Hispanic Asian	3192	3355 (3093 - 3639)	-163	-4.9	.186

Non-Hispanic Black	2868	3135 (2963 - 3317)	-267	-8.5	.002

Non-Hispanic Native Hawaiian	75	75 (43 - 130)	0	0.0	1.00

Non-Hispanic White	1829	1954 (1812 - 2108)	-125	-6.4	.085

Non-Hispanic Multiple/Unknown	99	55 (32 - 95)	44	80.0	.080

**Age at TB diagnosis in years**					

0-4	401	464 (405 - 532)	-63	-13.6	.040

5-14	245	292 (241 - 352)	-47	-16.1	.061

15-24	1274	1494 (1360 - 1641)	-220	-14.7	<.001

25-44	3893	4098 (3897 - 4309)	-205	-5.0	.048

45-64	3434	3846 (3624 - 4082)	-412	-10.7	<.001

65+	2292	2358 (2240 - 2482)	-66	-2.8	.339

Unknown	6	1 (1 - 1)	5	500.0	.041

**Disease location**					

Pulmonary only	7976	8751 (8436 - 9078)	-775	-8.9	<.001

Extrapulmonary only	2383	2594 (2465 - 2730)	-211	-8.1	.003

Both	1028	1169 (1031 - 1326)	-141	-12.1	.026

Unknown	158	27 (3 - 211)	131	485.2	.344

**Case verification**					

Positive culture	8876	9706 (9423 - 9997)	-830	-8.6	<.001

Positive NAA†	57	--	--	--	

Positive smear	91	63 (45 - 89)	28	44.4	.084

Clinical case	1630	1426 (1243 - 1637)	204	14.3	.048

Provider diagnosis	891	1364 (1246 - 1492)	-473	-34.7	<.001

**Homeless in the year** **prior to TB diagnosis**					

Yes	571	695 (623 - 774)	-124	-17.8	<.001

No	10797	11833 (11540 - 12133)	-1036	-8.8	<.001

Unknown	177	57 (22 - 147)	120	210.5	.094

**Resident of a correctional facility at time of TB diagnosis**					

Yes	461	480 (389 - 593)	-19	-4.0	.682

No	11041	12055 (11796 - 12319)	-1014	-8.4	<.001

Unknown	43	7 (2 - 29)	36	514.3	.189

**Excess alcohol use in the year prior to TB diagnosis**					

Yes	1382	1528 (1414 - 1652)	-146	-9.6	.013

No	9877	10889 (10641 - 11144)	-1012	-9.3	<.001

Unknown	286	180 (129 - 251)	106	58.9	.016

**Injecting drug use in the year prior to TB diagnosis**					

Yes	152	216 (182 - 256)	-64	-29.6	<.001

No	11087	12165 (11831 - 12510)	-1078	-8.9	<.001

Unknown	306	203 (129 - 320)	103	50.7	.093

**Non-injecting drug use in the year prior to TB diagnosis**					

Yes	828	922 (829 - 1026)	-94	-10.2	.046

No	10391	11438 (11178 - 11703)	-1047	-9.2	<.001

Unknown	326	221 (137 - 355)	105	47.5	.122

**HIV status at time of TB diagnosis**					

Positive	706	797 (748 - 849)	-91	-11.4	.005

Negative	6337	7621 (7171 - 8100)	-1284	-16.8	<.001

Unknown	4502	4347 (4134 - 4572)	155	3.6	.185

**Software for TB reporting**‡					

TIMS	4455	4775 (4552 - 5009)	-320	-6.7	.004

Other	7090	7771 (7470 - 8084)	-681	-8.8	<.001

*Expected case counts are derived from ratios (i.e., regression slopes on log-transformed observed counts). Because the sum of ratios is not the ratio of the sums, expected case counts stratified on case characteristics do not sum to overall case counts. All stratified count differences sum to within the 95% prediction interval (-688 to -1308) for the overall case count difference (-994).

† NAA = nucleic acid amplification test, which was not recorded as a TB case verification criteria until 2009, thus there are no comparable data from which to calculate expected values. In 2009, clinical cases include TB verified by positive interferon gamma release assay (IGRA) in lieu of tuberculin skin test, or by abnormal chest computerized tomography (CT) scan in lieu of chest radiograph; previously, such cases were verified by provider diagnosis because the National Tuberculosis Surveillance System did not collect IGRA or CT scan results prior to 2009.

‡TIMS = Tuberculosis Information Management System in operation since 1998. Other = National Electronic Disease Surveillance System-compliant software newly introduced in 2009.

### TB diagnostic and laboratory findings

We examined TB diagnostic characteristics to discover potential impacts of the revised 2009 TB case definition. Among cases in the NTSS, the majority (77%) in 2009 were verified by positive culture of *M. tuberculosis *(Table 1). Among persons aged 15 years and older with culture-positive pulmonary TB, there was a -14.2% decline from expected smear-positive cases and an -18.1% decline in cavitary disease, with similar declines among the U.S.-born and foreign-born. Unexpected declines were significant among both HIV-infected and uninfected persons (Table 1).

CDC-funded laboratories reported receiving clinical specimens from 103,708 individual patients in 2008 and 97,568 individual patients in 2009, a -5.9% decrease. In 2008, 4,972 (4.8%) patients had cultures positive for *M. tuberculosis *complex; this dropped to 4,217 (4.3%) in 2009, a decline of -9.8% in the proportion positive (*P *< .001).

### Surveillance reporting system and state-level analyses

Declines from expected counts were similar when comparing the 11 states that used the legacy electronic reporting system in 2009 (-6.7%) to the 39 states and Washington D.C. that used newly introduced software systems (-8.8%) [Table 1]. Compared to expected case counts, 31 states and Washington, D.C. had case declines; six of the 31 state counts were significantly lower than expected (Florida, Pennsylvania, North Carolina, Minnesota, Maryland, Michigan).

Of TB control program managers from 59 reporting areas, 55 responded to the National Tuberculosis Controllers Association survey (93%). We received no indication of changes in TB diagnostic or laboratory procedures. We examined the association of case count declines and staff reductions among 29 states reporting more than 100 cases in 2008. Unexpected declines were greatest in the states with no staff reductions (-21.2%), followed by states that lost 1-3 (-13.9%), more than 3 and fewer than 10 (-8.2%), or more than 10 (-6.1%) staff positions.

In the 10 counties and one city that matched TB case records across county, state, and national case reports, we did not find any cases unreported to CDC in 2008 (n = 2,940) or 2009 (n = 2,496). These jurisdictions reported the total number of culture-positive cases as 443 for the first quarter of 2008, decreasing to 344 cases for the first quarter of 2009, but for 2010, increasing to 379 cases in the first quarter.

### Time trends in TB treatment initiation and pharmacy sales

**Figure 2:**
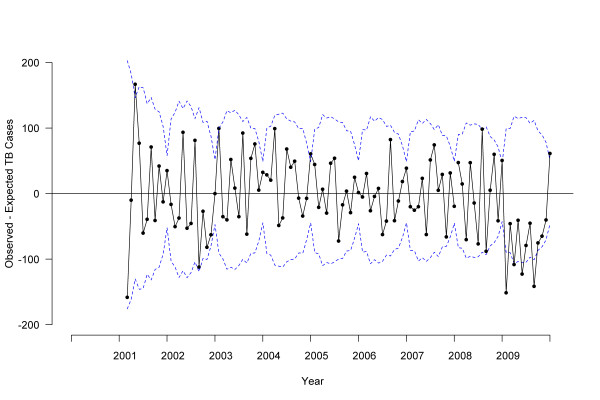
**Difference between observed and expected tuberculosis cases by treatment start date, 2000-2009, United States**. Solid line indicates the difference between observed and expected tuberculosis case counts based on autoregressive integrated moving average (ARIMA) model, dotted line indicates 95% confidence interval.

**Figure 3:**
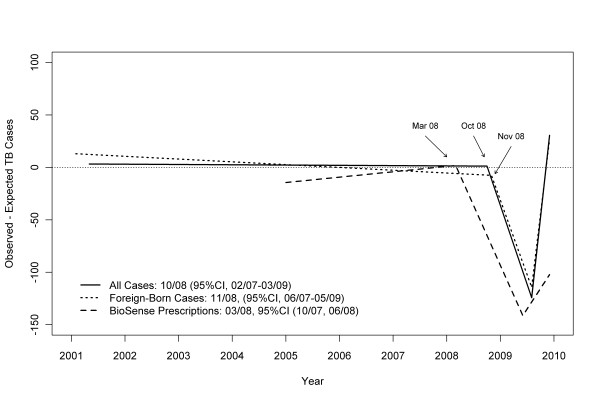
Tuberculosis trends for all cases and foreign-born cases* (National Tuberculosis Surveillance System) and outpatient pharmacy claims (BioSense). * U.S.-born cases showed significant decline in 2009 but analyses did not detect significant change attributed to a single flexion point in time

ARIMA analyses showed no difference between 2008 and 2001-2007 TB trends (*P *= .90), but did show significant declines for 2009 compared to 2008 (*P *= .01), and for 2009 compared to 2001-2007 (*P *= .005) [Figure 2]. Joinpoint analyses identified two significant changes: a decrease in TB cases starting in October 2008 (95% confidence interval [CI], February 2007 to March 2009) and an increase in August 2009 (95% CI, December 2008 to September 2009) [Figure 3]. Joinpoint analyses of BioSense TB prescription claims identified a decrease in March 2008 (95% CI, October 2007 to June 2008) and an increase in June 2009 (95% CI, September 2008 to September 2009) [Figure 3].

### TB time trends and rates among foreign-born and U.S.-born persons

**Figure 4:**
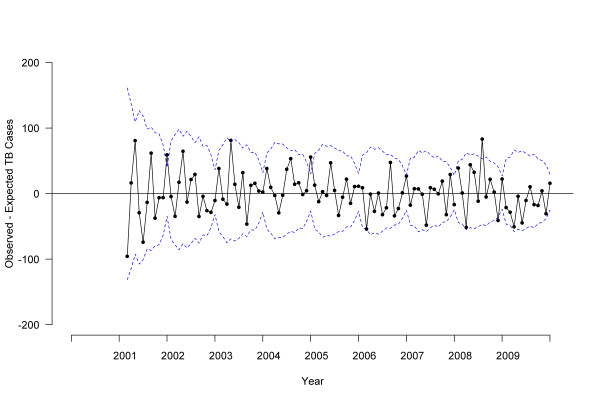
**Difference between observed and expected U.S.-born tuberculosis cases by treatment start date, 2000-2009, United States**. Solid line indicates the difference between observed and expected tuberculosis case counts based on autoregressive integrated moving average (ARIMA) model, dotted line indicates 95% confidence interval.

**Figure 5:**
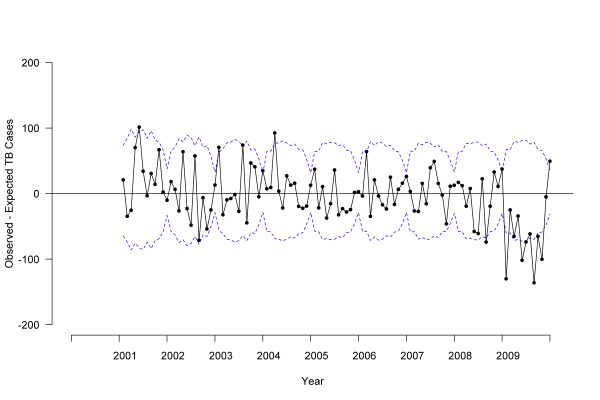
**Difference between observed and expected foreign-born tuberculosis cases by treatment start date, 2000-2009, United States**. Solid line indicates the difference between observed and expected tuberculosis case counts based on autoregressive integrated moving average (ARIMA) model, dotted line indicates 95% confidence interval.

Stratified ARIMA models indicated that case counts in 2009 were lower than expected among both U.S.-born (*P *= .04; Figure 4) and foreign-born (*P *= .008; Figure 5) persons. Joinpoint analyses failed to detect a significant change in monthly trend attributed to a single flexion point in time among U.S.-born persons, but did find a significant decrease among foreign-born persons beginning in November 2008 (95% CI, June 2007 to May 2009) [Figure 3]. When data for foreign-born persons were examined by time since U.S. arrival, a significant decrease was identified in October 2007 (95% CI, February 2007 to March 2008) among those with less than 2 years between arrival and TB treatment; however, no significant change point in time was identified for foreign-born persons with more than 2 years since arrival. Values of R^2 ^for the ARIMA models ranged from 0.93 to 0.96, indicating excellent model fit. No outliers were detected for foreign-born or overall TB cases, with three moderately outlying points detected for U.S.-born cases. After adjustment for outliers, the revised R^2 ^for U.S.-born changed from 0.93 to 0.94.

According to Census data, the population of U.S.-born persons in the United States increased 1.1% in 2009 compared with 2008; in contrast, the population of foreign-born persons fell 1.6% during this time. The rate of TB in 2009 among U.S.-born persons was 1.7 per 100,000, compared with an expected 1.9 per 100,000 persons (95% PI, 1.8 to 1.9). Among foreign-born persons, the rate of TB in 2009 was 18.7 per 100,000 persons, compared with an expected rate of 19.3 per 100,000 (95% PI, 18.8 to 19.9).

### TB transmission analyses

Among foreign-born persons, genotype clustering decreased during 2007-2008 (26.3% to 23.2%; *P *< .001) and 2008-2009 (23.2% to 20.2%; *P *< .001), while among the U.S.-born, clustering decreased from 2007-2008 (35.5% to 32.4%; *P *= .01) but not from 2008-2009 (32.4% to 31.2%; *P *= .36). Based on published ranges [14,15], we estimated the probability of transmission from a foreign-born source case to a U.S.-born secondary case as between 0.8% and 4.5%. The decline of -855 foreign-born cases (Table 1) might have resulted in between -38 to -6 fewer U.S.-born secondary cases.

## Discussion

We conclude that observed declines in 2009 TB case counts accurately reflect a true decline in the number of TB cases diagnosed in the United States, and that the deviation from past trends is significant. The unexpected decline in reported TB cases began rather abruptly in late 2008 or early 2009, and may have begun to reverse in mid 2009. We were able to exclude changes in electronic reporting systems as a causal factor, since declines were seen in states that used new software and well as states that did not. Independent information systems (NTSS, BioSense prescription claims, and public health laboratories) reported similar magnitude declines. Reductions in TB program staff were not associated with greater declines, and TB controllers reported no procedural changes. We found no cases unreported to the NTSS in our examination of over 5400 individual line-listed reports in 11 areas. A detailed study in two states similarly found no evidence of surveillance underreporting [16].

Declines were seen both in culture-positive TB cases and in cases lacking diagnostic laboratory findings. Analyses showed declines particularly among the foreign-born, and among Hispanics and non-Hispanic blacks. Declines were observed among foreign-born persons from countries that implemented new TB procedures for overseas screening and from countries using older procedures for pre-immigration screening. Updated pre-immigration screening to identify and treat TB prior to U.S. arrival is expected to reduce TB in the United States. A recent California study reported decreased TB incidence diagnosed within 6 months of U.S. arrival in persons screened with revised pre-immigration TB screening, compared to persons screened under the older procedures [17]. Authorized immigrants screened prior to U.S. arrival comprise approximately 500,000 arrivals in the United States each year, compared to greater than 160 million annual nonimmigrant admissions among students, temporary workers, diplomats, family members, and visitors, who are not routinely screened prior to U.S. arrival [18]. Because immigration status was not available for analysis of NTSS data, we were not able to adjust for whether foreign-born persons diagnosed with TB in the United States were screened with the revised or older pre-immigration procedures, only whether they originated from a country that had begun to implement updated procedures prior to 2009.

The unexpected decline in TB cases among the foreign-born could have occurred because of fewer persons entering or more persons leaving the United States. U.S. Census figures estimate a -1.6% decline in the foreign-born population from 2008 to 2009. Since there was an -11.1% decline in observed compared with expected foreign-born TB cases, the decline in population does not explain the entire decline in foreign-born TB case counts. Our finding that the decline in foreign-born cases was most prominent among those whose TB occurred within 2 years of their arrival, and among persons from Mexico and Guatemala (who together comprised 26% of foreign-born cases), might indicate that the March 2009 Census population data do not accurately reflect population changes in these groups. Department of Homeland Security data suggest greater declines in the foreign-born population in the United States than do Census data, including a -6.9% decrease from 11.6 to 10.8 million unauthorized immigrants from 2008 to 2009 [19]. Our finding is consistent with analyses showing that the decline in unauthorized immigrants in the United States is strongly associated with decreased immigration from, and increased deportation to, Mexico [20]. Nonimmigrant authorized admissions to the United States also declined, from 175.4 million in 2008 to 162.6 million in 2009 [18]. Financial data indicate that remittances to Mexico and Latin America began to decline in 2008 and early 2009, consistent with the timing of TB declines, and began to rebound in late 2009 [21].

Case count declines among the U.S.-born were less impressive than among the foreign-born and cannot be substantially explained by less TB transmission from fewer foreign-born cases or by less transmission among the U.S.-born, since genotype clustering among the U.S.-born did not significantly decline from 2008 to 2009. A limitation of this analysis is that, although genotype clustering is thought to be associated with recent transmission, it is not a direct measure of transmission. Nevertheless, assessment of clustering did not show an abrupt decline in genotype clustering coincident with the abrupt decline in TB cases, suggesting that the decline in cases was not the result of a sudden improvement in TB control practices.

Some of the most dramatic percentage declines occurred among minorities and the socially disadvantaged. These declines may reflect a general problem of delayed access to diagnostic services related to the recent economic recession and some combination of loss of health insurance, fear that seeking medical care might lead to legal consequences, or inability to pay for services [22-24]. We were not able to assess health insurance status or health seeking behavior among TB patients; however, recent national reports of overall declines in physician visits [25] and correlation between unemployment and TB incidence [26] suggest that the decline in reported TB cases may be further evidence of the impact of the economic recession.

Our finding that laboratory reports of culture-confirmed diagnoses of TB as a proportion of specimens submitted declined more (-9.8%) than did the number of patients who had specimens submitted for culture (-5.9%) is not consistent with a decline in the index of suspicion for TB among healthcare providers. The abruptness of the decline in cases beginning in late 2008 or early 2009 is also not consistent with this hypothesis.

Although we ruled out surveillance artifact in terms of the total case count, there were increases in missing data in some routinely collected variables, representing small numbers but large increases compared to expectation, which could affect the interpretation of our analyses. For example, if missing data for social risk factors such as injecting drug use and homelessness were complete, our finding of steep declines among persons with these risk factors might either remain unchanged (if missing responses were "no," or were distributed proportional to observed data) or be attenuated to indicate lesser declines (if missing social risk factors were "yes"). Typically, the national surveillance database becomes more complete over time, since TB programs have two years to follow and close out cases in electronic reporting. This incompleteness represents a limitation to using surveillance data for our comparison of observed case counts to expected counts based on prior year counts, which are more complete.

In 2010, provisional NTSS data include 11,181 TB cases reported in the United States, for a rate of 3.6 cases per 100,000 population, which was a decline of -3.9% from 2009 similar to the average decline in TB rates (-3.8% per year) from 2000-2008 [27]. Although the steady decline in TB rates in the United States is evidence of continued progress in TB control, the abrupt decline in 2009 followed by a return to average decline is not consistent with sudden improvements in TB control efforts. Multiple causes coincident with economic recession in the United States, including decreased immigration and delayed access to medical care, could be related to TB declines.

## Conclusion

While a greater-than-expected decline in a deadly infectious disease is usually reason for celebration, our results suggest caution. To the extent that the 2009 TB decline was due to change in immigration patterns, we might expect future increases as the economy improves and the immigrant population increases [28-30]. To the extent that declines represent delays in accessing health care, there might be a greater than expected number of future TB diagnoses, and future cases may be more advanced, more infectious, and more difficult to cure [31]. Ecologic data suggest that rebounds in infectious diseases after economic disruption can have long-standing consequences [32]. To achieve TB elimination in the United States, public health programs must address conditions associated with TB and with disparities in obtaining health services [33].

## Competing interests

The authors have no competing interests to report. The findings and conclusions are those of the authors and do not necessarily represent the views of the Centers for Disease Control and Prevention.

## Authors' contributions

CAW had full access to all of the data in the study and takes responsibility for the integrity of the data and the accuracy of the data analysis. CAW drafted the manuscript, with sections contributed by TRN, JEB, MPC, AMS, CH, and JSK. CAW, JEB, and MPC designed the overall statistical analysis plan. CAW, TRN, LRA, CJ, RSYW, JW, AMS, CMH, JSK, MCM, SP, JS, AMC, and PG collected and analyzed data and reviewed the findings for interpretation and manuscript for critical intellectual content. CAW, TRN, JEB, LRA, JW, WRM, KM, PAL, and KGC contributed to the study design and interpretation of data as well as critical review and editing of the manuscript. All authors read and approved the final manuscript.

## Pre-publication history

The pre-publication history for this paper can be accessed here:

http://www.biomedcentral.com/1471-2458/11/846/prepub
